# Dnmt3a in the dorsal dentate gyrus is a key regulator of fear renewal

**DOI:** 10.1038/s41598-018-23533-w

**Published:** 2018-03-23

**Authors:** Zhiting Gong, Qiang Zhou

**Affiliations:** School of Chemical Biology and Biotechnology, Peking University Shenzhen Graduate School, Shenzhen, China

## Abstract

Renewal of extinguished fear memory in an altered context is widely believed to be a major limiting issue for exposure therapy in treating various psychiatric diseases. Effective prevention of fear renewal will significantly improve the efficacy of exposure therapy. DNA methyltransferase (DNMTs) mediated epigenetic processes play critical roles in long term memory, but little is known about their functions in fear memory extinction or renewal. Here we investigated whether DNMTs regulate fear renewal after extinction. We found that elevated Dnmt3a level in the dorsal dentate gyrus (dDG) of hippocampus was associated with the absence of fear renewal in an altered context after extinction training. Overexpression and knockdown of Dnmt3a in the dDG regulated the occurrence of fear renewal in a bi-directional manner. In addition, Dnmt3a overexpression was associated with elevated expression of c-Fos in the dDG during extinction training. Furthermore, we found that renewal of remote fear memory can be prevented, and the absence of renewal was concurrent with an elevated Dnmt3a level. Our results indicate that Dnmt3a in the dDG is a key regulator of fear renewal after extinction, and Dnmt3a may play a critical role in controlling fear memory return and thus has therapeutic values.

## Introduction

Exposure therapy has widely been used and is effective in treating various psychiatric disorders, especially phobia and PTSD. Clinical literature shows that fear may return with the passage of time or in a context distinct from the treatment context, after therapy^1,2^. The return of fear in an altered context is termed fear renewal. One possible cause of fear return is change of the post-treatment context, and this notion is supported by clinical demonstrations that entering a new context promotes fear return^3–6^. Therefore, preventing the return of fear in the post-exposure contexts, especially those distinct from the treatment/therapy context, is a critical obstacle to higher efficacy of exposure therapy.

Pavlovian fear conditioning and extinction is a widely used experimental model to study the biological mechanisms underlying exposure therapy. Most studies have shown fear extinction in the adult rodents to be context-dependent, using a standard extinction protocol (usually continuous presentation of conditioned stimulus (CS))^7,8^. Recent studies showed that a brief memory retrieval followed by extinction training led to an extinction that is insensitive to renewal, spontaneous recovery and reinstatement in both rodents^9–11^ and human^12^. However, the reproducibility of these findings has been questioned by recent studies^13–15^. More importantly, the mechanism of preventing fear renewal is not well understood.

Persistent and long-term changes in neuronal structure and function, such as those occurring during memory formation, generally require epigenetic modifications. Numerous studies have demonstrated that alterations in histone acetylation and DNA methylation are involved in the formation and extinction of long-term memory^16–19^. DNMTs (Dnmt1, Dnmt3a, Dnmt3b and Dnmt3l) catalyze the cytosine methylation and are required to establish and maintain genomic methylation. Dnmt3a and Dnmt3b are de novo DNA methyltransferases, Dnmt1 is the maintenance DNA methyltransferase, while Dnmt3l has no DNA methyltransferases activity^20^. Knockout restricted to the forebrain excitatory neurons in mice demonstrated learning deficits in several associative and episodic memory tasks in Dnmt3a knockout but not Dnmt1 knock out^21^. Knockdown of Dnmt3a but not Dnmt3b in hippocampus resulted in dysfunction of object-in-place memory^22^. Knocking down Dnmt3a in the medial prefrontal cortex increased anxiety while overexpression reduced anxiety^23^. Dnmt3a has also been shown to regulate emotional behavior and dendritic spine plasticity in the nucleus accumbens^24^. Thus, Dnmt3a plays important functions in neural plasticity, long-term modifications and emotional functions.

In this study, by using fear renewal in an altered context as readout, we found that Dnmt3a expression was elevated in the dorsal dentate gyrus (dDG) after extinction training followed by a brief memory retrieval (Rec+Ext), which was associated with the absence of fear renewal when tested in an altered context. Increasing Dnmt3a expression in the dDG using AAV expression led to the prevention of fear renewal following a standard extinction training protocol. Knockdown of Dnmt3a in the dDG using CRISPR/Cas9 resulted in fear renewal following Rec+Ext protocol. Furthermore, we found that renewal of remote fear memory can be prevented using the Rec+Ext protocol, and the absence of renewal was concurrent with an elevated Dnmt3a level.

## Result

### Elevated Dnmt3a expression associated with the absence of fear renewal after extinction

**Figure 1:**
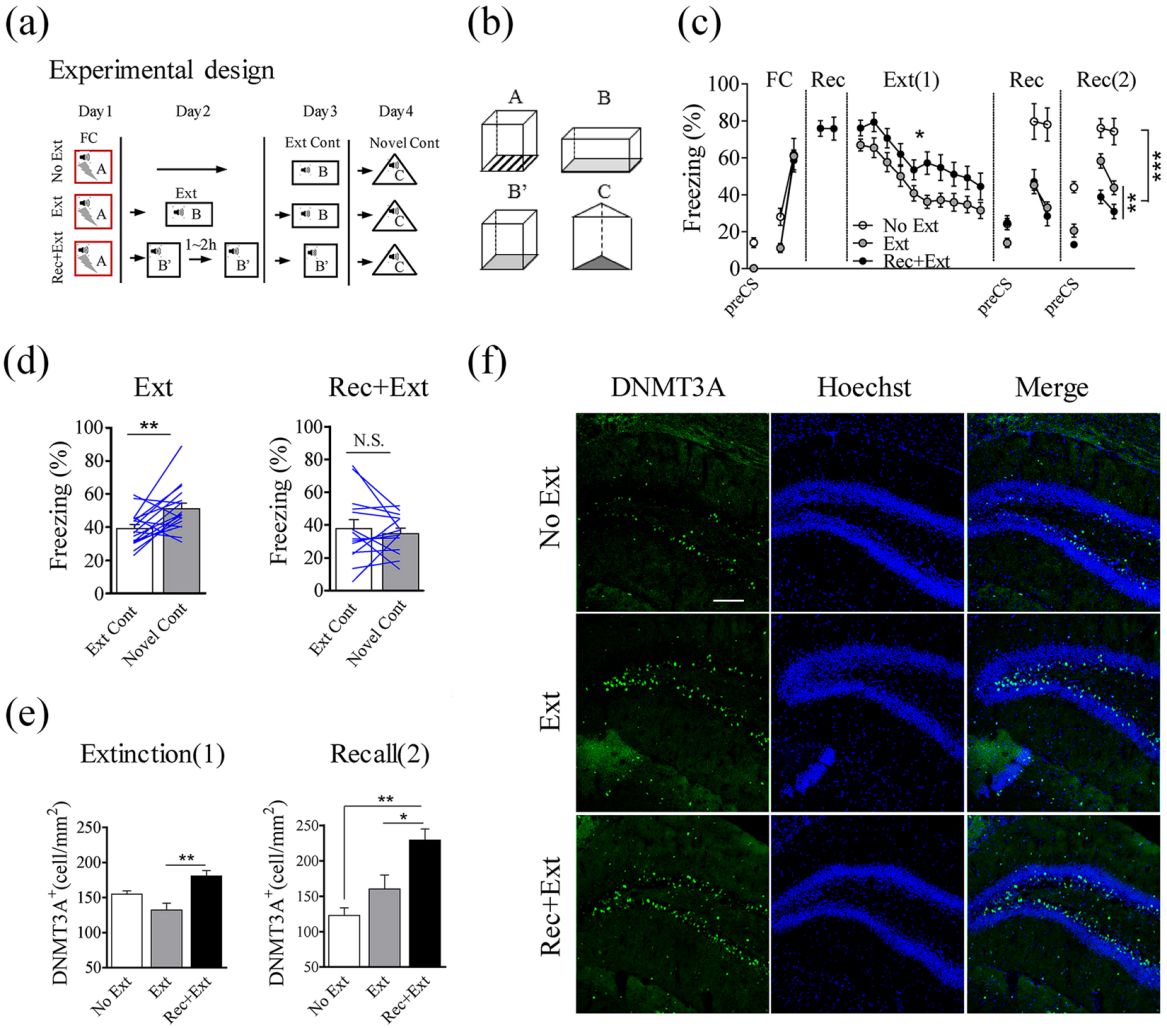
Elevated density of Dnmt3a-positive cells in the hippocampal dorsal DG associated with the absence of fear renewal. (**a**) Experimental procedures. Day 1, fear-conditioning (FC); Day 2, a standard extinction training (Ext, n = 17 mice), a brief recall followed by extinction training (Rec+Ext, n = 14), or no extinction training (No Ext, n = 5); Day 3, testing in extinction context (B or B’); Day 4, testing in a novel context (C). (**b**) Four distinct contexts used. (**c**) Freezing levels during the entire experimental course (averages during two CS). Significant difference was found between Ext and Rec+Ext groups during extinction training (Two-way ANOVA, repeated measures (RM), F_1,261_ = 7.35, P < 0.05) and during Recall in context C (Two-way ANOVA, RM, F_1,29_ = 12.7, P < 0.01). (**d**) Freezing levels (average during four CS) of individual mouse during recall in the extinction and novel context. Significant difference was found between these two contexts for Ext group (left; Two-tailed paired *t*-test, T_16_ = 3.20, P < 0.01), but not for Rec+Ext group (right, Two-tailed paired *t*-test, T_13_ = 0.61, P = 0.55). (E) (Left) Significant elevation in the density of Dnmt3a-positive cells in the Rec+Ext group, compared to Ext group (n = 4 mice, at least 3 sections per mouse; One-way ANOVA followed by Tukey’s post hoc test, F_2,9_ = 9.95, P < 0.01). Samples were collected 3 hours after Ext or Rec+Ext (marked as “1” corresponding to Ext(1) in C), and No Ext group collected 3 hours after recall in context B’ on Day 2. (Right) Significant differences were found between Ext and No Ext groups, and between Ext and Rec+Ext groups (n = 5 mice, at least 3 sections per mouse; One-way ANOVA followed by Tukey’s post hoc test, F_2,12_ = 11.74, P < 0.05). Samples were collected 3 hours after recall in context C (marked as “2” corresponding to Rec(2) in C). (**f**) Sample images of Dnmt3a staining in the dDG after extinction training, collected 3 hours after recall in context C. Scale bars, 100 μm. Data are presented as mean ± SEM; *P < 0.05; **P < 0.01; ***P < 0.001; N.S., Not significant.

We modified the protocol from Monfils (2009)^9^, which consisted of a brief fear memory recall followed (in 1–2 hours) by 20 cycles of CS (termed Rec+Ext) (Fig. 1a). It has been shown that if extinction context is very distinct from the conditioning context, the Rec+Ext protocol did not prevent renewal of fear memory^14^. Thus, we used a Context B’ modified from the original fear conditioning chambers (see Methods) (Fig. 1b). A standard protocol with continuous 20-cycle CS presentation (termed Ext; Fig. 1a) was used in context B (see Methods) (Fig. 1b) which has been shown by many previous studies to lead to fear renewal after extinction. After fear conditioning (Day 1), mice were divided into three groups with similar freezing levels: no extinction training (termed No Ext), Ext or Rec+Ext. Day 2, the No Ext group did not receive any extinction training, while the Ext group received 20 cycles CS training in context B and the Rec+Ext group first received 4 cycles CS training in context B’ followed in 1~2 hours with 20 cycles CS training in context B’. All mice were tested in the extinction context (B/B’; after 24 hours, or on Day 3) and novel context (C; after 48 hours, or on Day 4) (Fig. 1a). The Ext and Rec+Ext groups exhibited similar freezing levels after fear conditioning and showed significant reduction in freezing during extinction training (Fig. 1c). The Rec+Ext group showed higher freezing level compared to the Ext group during extinction training (Fig. 1c), and this higher freezing level could be caused by the similarity between context B’ and conditioning context A. Although recall in the extinction context revealed no difference between these two groups, Ext group showed significantly higher freezing than the Rec+Ext group in novel context (C) (F_1,29_ = 12.7, P < 0.01, Fig. 1c). The Ext group showed higher freezing level in context C as compared to context B (T_16_ = 3.20, P < 0.01, Fig. 1d left), while the Rec+Ext group showed similar freezing level in these two contexts (Fig. 1d right).

Previous studies have shown that Dnmt3a is intimately involved in regulating the stable expression of fear memory^21^, thus we examined whether Dnmt3a level is altered using immunostaining in brain sections. Mouse brains were collected 3 hours after extinction training (Ext or Rec+Ext) on Day 2, or 3 hours after 4 cycles CS recall in context C on Day 4. Higher density of Dnmt3a-positive cells was observed in the dDG in Rec+Ext group (which showed an absence of renewal) compared to the Ext group, using brains collected either after extinction training (F_2,9_ = 9.95, P < 0.01, Fig. 1e left) or after recall in context C (F_2,12_ = 11.74, P < 0.05, Fig. 1e right, Fig. 1f). These results suggest an association between Dnmt3a expression and fear memory renewal in that elevated Dnmt3a level may prevent fear renewal.

### Overexpression of Dnmt3a in the dDG prevented fear renewal after extinction

**Figure 2:**
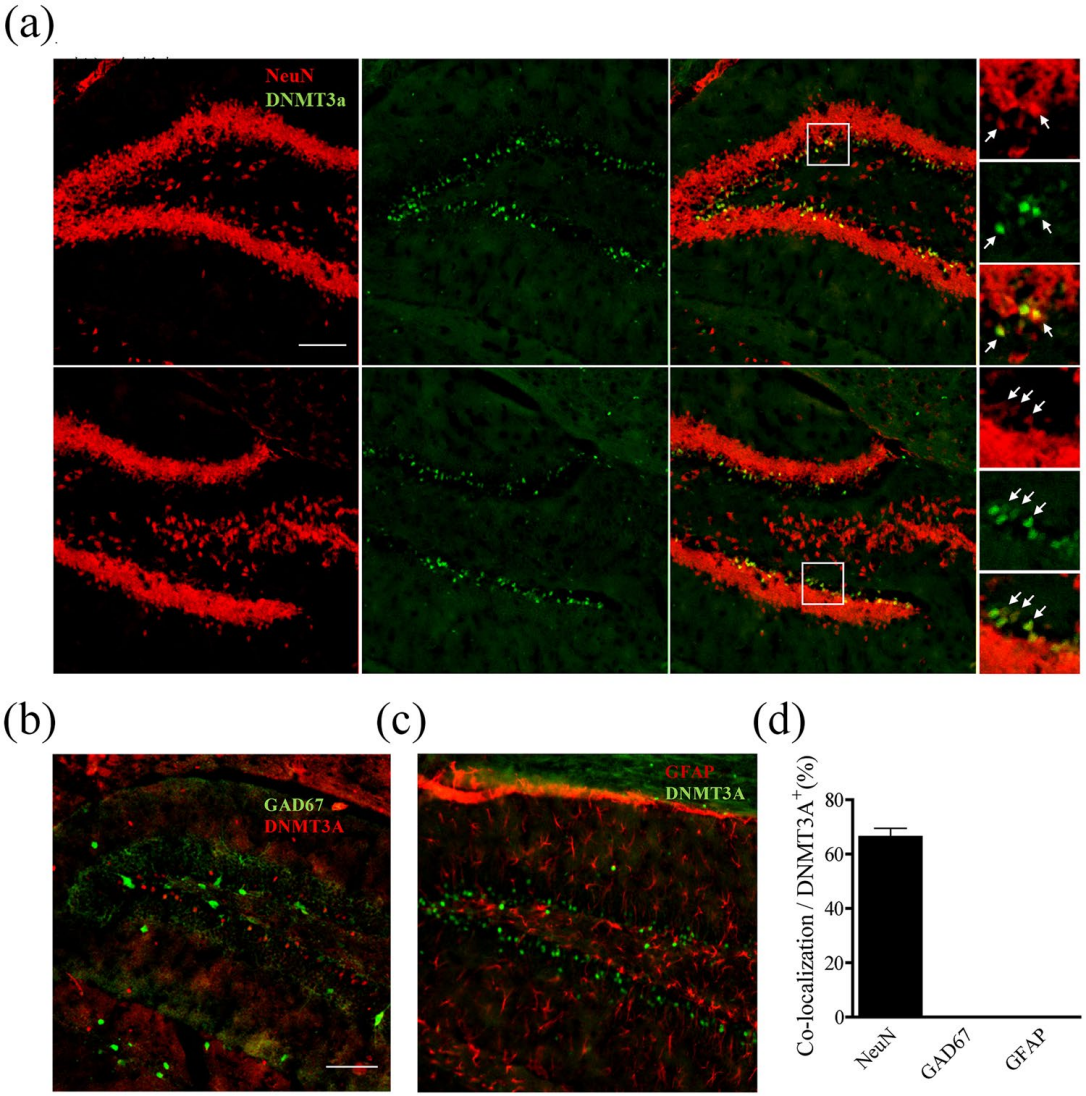
Cell types that showed elevated Dnmt3a expression. Double immunostaining of Dnmt3a and NeuN (**a**), Dnmt3a and GAD67 (using GAD67-GFP mouse) (**b**), Dnmt3a and GFAP (**c**). Regions inside the boxes were enlarged on the right showing red, green and merge. Co-localized spots were marked by arrows. (**d**) Higher than 70% of Dnmt3a-positive cells were also NeuN positive (n = 5 mice). Scale bars, 100 μm.

**Figure 3:**
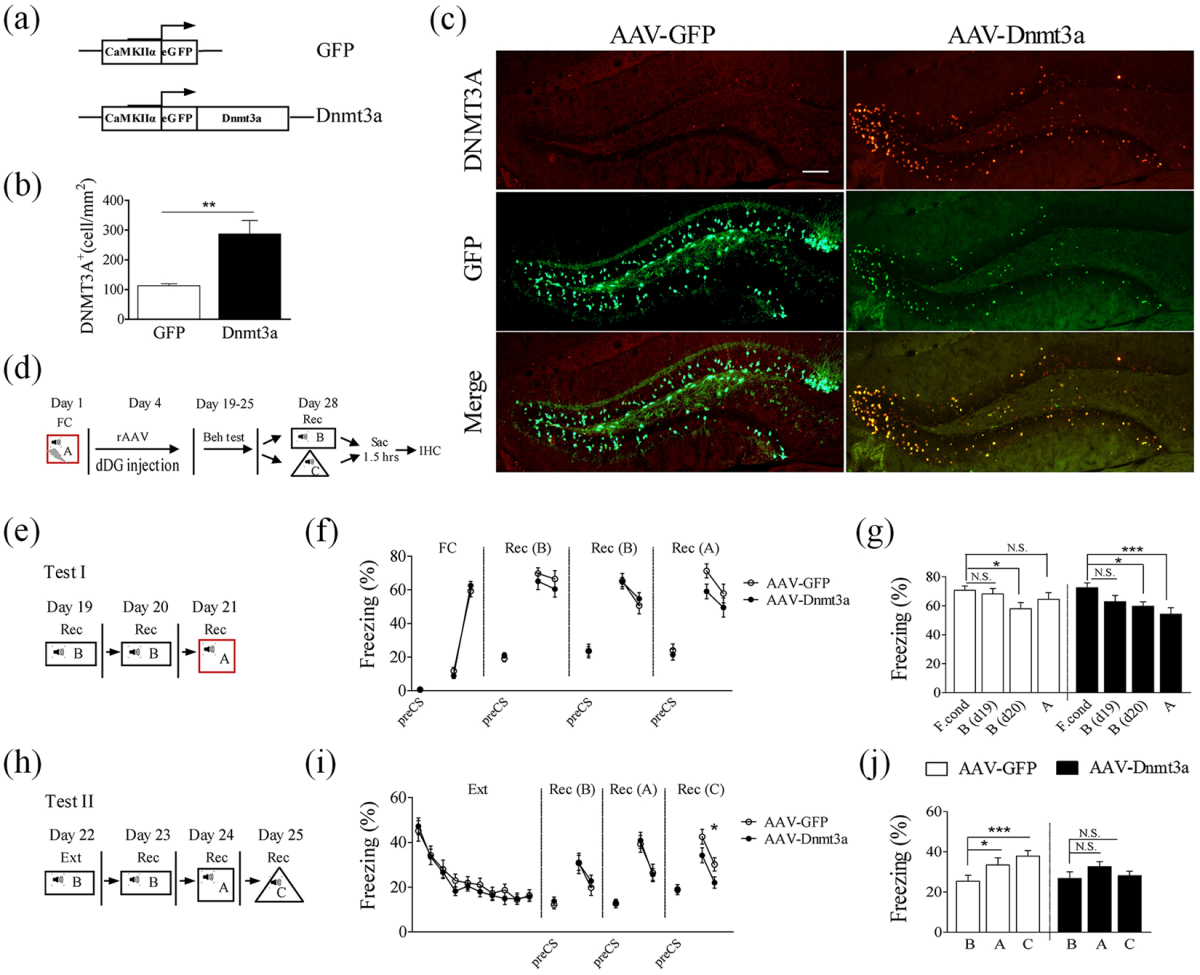
Dnmt3a overexpression in the dorsal DG prevented fear renewal after standard extinction training. (**a**) The viral constructs used. (**b**) Significantly higher Dnmt3a-positive cells in the dDG of mice injected with AAV-Dnmt3a (n = 6 mice), compared to AAV-GFP (n = 6) (Two-tailed unpaired *t*-test, T_10_ = 3.84, P < 0.01). (**c**) Sample images. Scale bar, 100 μm. (**d**,**e**) Experimental designs. (**f**) Freezing level (averages during two CS) of the entire experiment. (**g**) Behavioral results. F. Cond, freezing levels of the last CS-US pairing; B (d19), B (d20) and A, averaged freezing levels during four CS during recall. Two-way ANOVA, RM with Bonferroni post-test, compare B (19), B (20) and A to F. Cond. AAV-Dnmt3a [compare B (19), B (20) and A to F. Cond], B (d19) vs. F. Cond, t_20_ = 2.010, P > 0.05; B (d20) vs. F. Cond, t_20_ = 2.660, P < 0.05; A vs. F. Cond, t_20_ = 3.779, P < 0.001. AAV-GFP [compare B (19), B (20) and A to F. Cond], B (d19) vs. F. Cond, t_22_ = 0.544, P > 0.05; B (d20) vs. F. Cond, t_22_ = 2.766, P < 0.05; A vs. F. Cond, t_22_ = 1.324, P > 0.05). AAV-Dnmt3a group (n = 21 mice), GFP group (n = 23 mice). (**h**) Experimental designs. (**i**) Freezing level for test II. There was a significant difference between two groups (Two-way ANOVA, RM, F_1,42_ = 5.15, P < 0.05). (**j**) Behavioral result of test II. Freezing levels were averages during four CS. For AAV-Dnmt3a group, no significant difference in freezing levels among context B, A and C. For AAV-GFP group, freezing level in context A or C was significantly higher than that in context B (Two-way ANOVA, RM with Bonferroni post-test, compare A and C to B. AAV-Dnmt3a [compare A and C to B], A vs. B, t_20_ = 1.518, P > 0.05; C vs. B, t_20_ = 0.3663, P > 0.05. AAV-GFP [compare A and C to B], A vs. B, t_22_ = 2.397, P < 0.05; C vs. B, t_22_ = 4.064, P < 0.001).

To determine whether Dnmt3a regulates fear renewal, we need to target the cells that show changes in Dnmt3a. We found that a large percentage of the Dnmt3a-positive cells were co-localized with NeuN, a neuronal marker (Fig. 2a–d), but not co-localized with GAD67 (a GABA-synthesizing enzyme and a marker of inhibitory neurons) (Fig. 2b–d), or GFAP (a marker of astrocytes) (Fig. 2c,d). Based on this finding, we generated recombinant adeno-associated virus (rAAV) containing eGFP-tagged Dnmt3a under the CaMKIIα promoter to selectively target excitatory neurons (Fig. 3a). Expression of AAV for 14 days led to over-expression of Dnmt3a in the excitatory neurons in the dDG (T_10_ = 3.84, P < 0.01, Fig. 3b,c).

After fear conditioning, mice were divided into two groups with comparable freezing levels. Seventy-two hours after conditioning, mice were injected with either AAV-GFP or AAV-Dnmt3a in the dDG. Behavioral testing was conducted after rAAV expression for 14 days, with mouse brains collected 90 min after recall in context B/C (Fig. 3d). We first measured freezing levels, twice in context B and once in context A (Fig. 3e Test I). There was no difference in freezing level between two groups (Fig. 3f). Since Morris *et al*.^21^, found that Dnmt3a CKO mice showed a deficit in the reduction of cued fear memory upon repeated testing; we wanted to test whether Dnmt3a over-expression in the dDG might lead to faster fear memory decay/reduction with repeated recall. We found no difference in freezing levels in context B on day 19 (both were not significant from the freezing levels after fear conditioning), or on day 20 (both were significantly different; AAV-Dnmt3a, T_20_ = 2.660, P < 0.05; AAV-GFP, T_22_ = 2.766, P < 0.05; Fig. 3g). In contrast, AAV-Dnmt3a group showed significantly lower freezing level in context A compared to that after fear conditioning on day 21 (T_20_ = 3.779, P < 0.001, Fig. 3g), but no such difference was found in the AAV-GFP group (Fig. 3g). These results suggest that overexpression of Dnmt3a appears to mask the effect of context on memory recall.

We then asked whether fear renewal could be blocked or prevented with the Ext protocol. Mice received Ext training in context B on day 22, and freezing levels were measured in the extinction context (B) on day 23, in conditioning context (A) on day 24 and in novel context (C) on day 25 (Fig. 3h, Test II). There was no difference in freezing levels between the AAV-Dnmt3a group and AAV-GFP group during extinction training, recall in the extinction context (B) and conditioning context (A), but there was a significant difference in recall in novel context (C). (F_1,42_ = 5.15, P < 0.05; Fig. 3i). In the AAV-Dnmt3a group, freezing levels in the above three contexts did not differ significantly from each (Fig. 3j). In contrast, AAV-GFP group showed significantly higher freezing in conditioning context (A) (T_22_ = 2.397, P < 0.05, Fig. 3j) and novel context (C) (T_22_ = 4.064, P < 0.001, Fig. 3j) than in the extinction context (B). Thus, fear renewal was absent with elevated Dnmt3a level in the dDG excitatory neurons. Dnmt3a has been reported to affect anxiety-like behavior^23^, and thus the effects on fear extinction and renewal we observed may be caused by altered innate anxiety level in the mice^25,26^. To address this possibility, we tested the performance of mice in the open field test and elevated plus maze. No significant difference in these two tests (such as percentage center time, open arm time) was found between the AAV-Dnmt3a and AAV-GFP group, with a trend towards increased total distance in the open field test (Fig. S1)

### Dnmt3a overexpression affected neuronal activity

**Figure 4:**
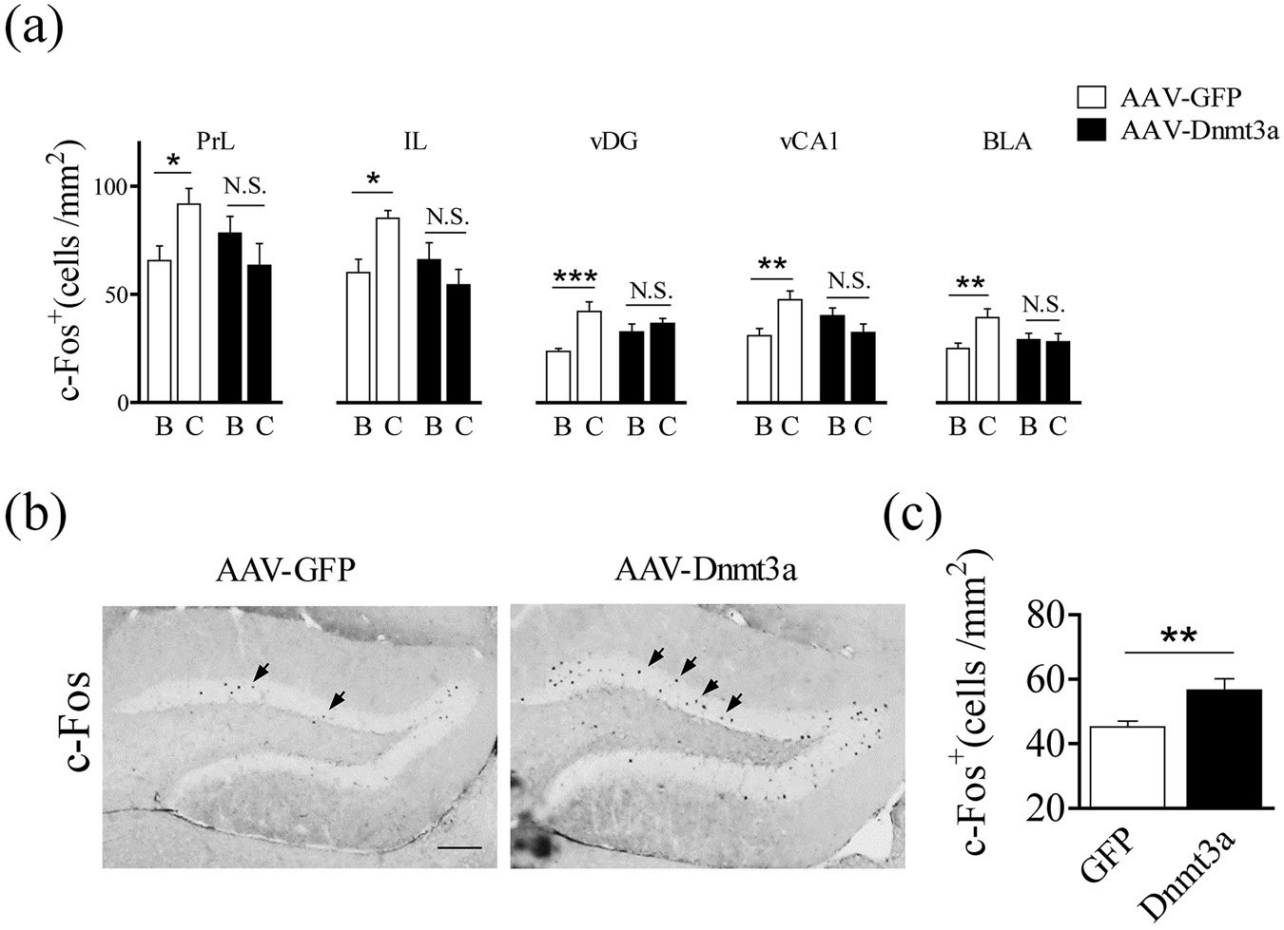
Patterns of c-Fos expression with overexpression of Dnmt3a in the dDG. (**a**) Brain sections were collected after recall in context B (AAV-GFP, n = 12 mice; AAV-Dnmt3a, n = 10 mice), recall in context C (AAV-GFP, n = 10 mice; AAV-Dnmt3a, n = 10 mice). AAV-GFP group showed elevated density of c-Fos cells in PrL (T_20_ = 2.35, P < 0.05), IL (T_20_ = 2.82, P < 0.05), vDG (T_20_ = 4.32, P < 0.001), vCA1 (T_20_ = 3.27, P < 0.01), BLA (T_20_ = 3.13, P < 0.01). No difference was seen after recall in context B or C in AAV-Dnmt3a group. PrL (T_18_ = 1.29, P > 0.05), IL (T_18_ = 1.26, P > 0.05), vDG (T_18_ = 0.89, P > 0.05), vCA1 (T_18_ = 1.48, P > 0.05), and BLA (T_18_ = 0.22, P > 0.05). Two-way ANOVA RM with Bonferroni post-test, B vs. C. (**b**) Sample images of c-Fos staining in DG. c-Fos positive cells were marked by arrows. Samples were collected after extinction training. Scale bars, 100 μm. (**c**) Density of c-Fos positive cells in the dDG. Sections were collected after extinction training in context B (AAV-GFP, n = 11 mice; AAV-Dnmt3a, n = 10 mice). Significant difference was seen between AAV-Dnmt3a and AAV-GFP group (Two-tailed unpaired *t*-test, T_19_ = 2.97, P < 0.01).

As the first step to understand the mechanism underlying the prevention of fear renewal in mice with overexpression of Dnmt3a in the dDG, we measured c-Fos expression in key brain regions involved in fear memory renewal. Numerous brain structures, including hippocampus, prefrontal cortex and amygdala participate in fear memory renewal^27^. We collected mouse brains 90 min after recall in context B or C on day 28 (Fig. 3d, behavioral results before c-Fos staining were shown in Fig. S2). AAV-Dnmt3a group showed similar density of c-Fos-positive cells in context B and C in vCA1, prelimbic PFC (PrL), infralimbic PFC (IL) and BLA (Fig. 4a), consistent with similar fear state in these mice. In contrast, AAV-GFP group showed elevated density of c-Fos cells in context C in PrL (T_20_ = 2.35, P < 0.05), IL (T_20_ = 2.82, P < 0.05), vDG (T_20_ = 4.32, P < 0.001), vCA1 (T_20_ = 3.27, P < 0.01), and BLA (T_20_ = 3.13, P < 0.01) (Fig. 4a), consistent with high fear state under this condition. AAV-GFP and AAV-Dnmt3a groups showed no difference in dHPC or vCA3 (Fig. S3). These results indicate that over-expression of Dnmt3a removes the context difference on neuronal activity level in key brain regions that regulate fear renewal^28,29^.

Since we had observed elevated Dnmt3a expression with Rec+Ext protocol which prevented fear renewal, we asked whether the activity level in the dDG might be altered during extinction training. To do so, we analyzed c-Fos expression in mouse brains collected 90 min after Ext training on day 22 (behavioral results shown in Fig. S4). The AAV-Dnmt3a group had higher density of c-Fos-positive cells than AAV-GFP group in the dDG (Fig. 4b; T_19_ = 2.97, P < 0.01, Fig. 4c), and there was a significant correlation between the density of c-Fos-positive and Dnmt3a-positive cells in the AAV-Dnmt3a group (Fig. S4). Due to technical reasons, we were unable to perform the same analysis for the AAV-GFP group. Furthermore, we examined the density of c-Fos-positive cells in mice received Rec+Ext protocol (which showed high Dnmt3a level in Fig. 1e left), and found higher density in the dDG compared to mice received the Ext protocol (Fig. S5). Put together, these results suggest that Dnmt3a overexpression in the dDG is associated with elevated neural activity in the dDG during extinction training which may serve to prevent fear renewal.

### Knockdown of Dnmt3a in the dDG promoted fear renewal

**Figure 5:**
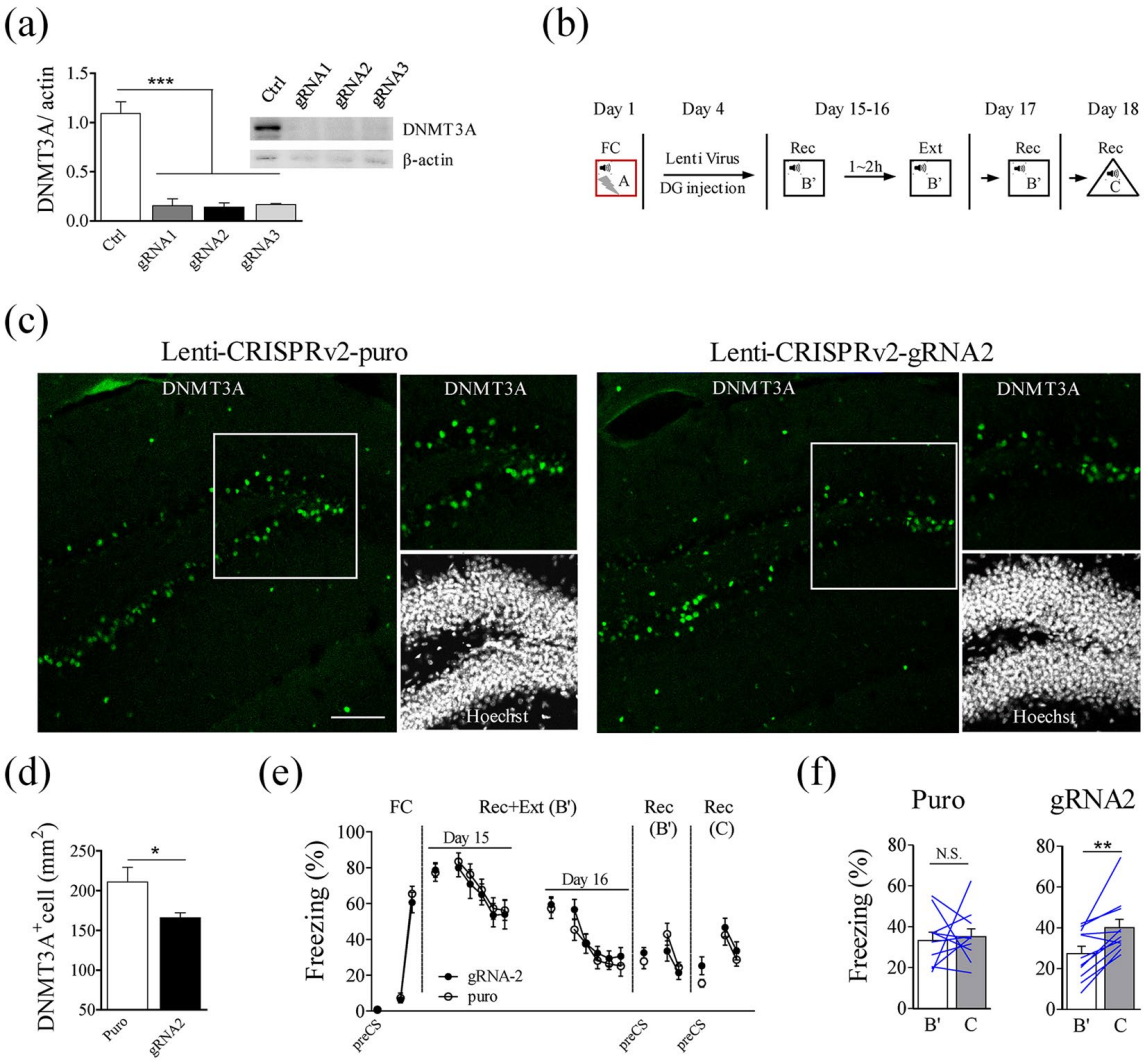
Knockdown of Dnmt3a in the dDG lead to fear renewal after extinction training with Rec+Ext protocol. (**a**) Western blot AAV-Dnmt3a group showed significantly reduced Dnmt3a level in lenti-CRISPRv2-gRNAtransfeced cells (One-way ANOVA, all columns vs. control, F_3,8_ = 42.27, p < 0.0001). Cells were collected 48 hours after co-transfection with plenti-CRISPRv2-gRNA/control and pcDNA3.0-Dnmt3a into HEK293 cells. (**b**) Experimental design for behavioral test. (**c**) Sample images of Dnmt3a staining in the dDG, injected with either lenti-CRISPRv2-gRNA2 or lenti-CRISPRv2-puro, and allowed to express for 10 days. Scale bars, 100 μm. (**d**) Significantly lower density of Dnmt3a-positive cells was observed in the dDG in lenti-CRISPRv2-gRNA2 injected group (n = 6 mice), compared to lenti-CRISPRv2-puro (n = 6 mice) (Two-tailed unpaired *t*-test, T_10_ = 2.27, P < 0.05). Samples were collected 10 days after injection. (**e**) Freezing levels during the entire experiment as averages during two CS presentations. Rec+Ext training on Day15–16 were averages during four CS presentations. (**f**) Freezing levels of individual mouse were shown for recall in context B’ and context C. In lenti-CRISPRv2-puro group, there was no significant difference between freezing levels during recall in context B’ and C (n = 10 mice, Two-tailed paired *t*-test, T_9_ = 0.30, P = 0.77). In lenti-CRISPRv2-gRNA2 group, freezing level in context C was significantly higher than that in context B’ (n = 11 mice, Two-tailed paired *t*-test, T_10_ = 4.04, p < 0.01). Freezing levels were average during four CS presentations.

To test whether Dnmt3a can regulate fear renewal in a bi-directional manner, we used CRISPR/Cas9 method to reduce Dnmt3a expression in the dDG^30^. We tested three guide RNA sequence (Supplementary Table) for constructing lenti-CRISPRv2 guide RNA vector, the efficiency of guide RNA was tested by transfecting HEK293T cells (Fig. 5a, Fig. S6). We packaged lentivirus using pLenti-CRISPRv2-gRNA2. Lenti-CRISPRv2-gRNA2 was able to significantly decrease Dnmt3a level in the dDG 10 days after infection (Fig. 5c; T_10_ = 2.27, P < 0.05, Fig. 5d). After fear conditioning on day 1, mice were divided into two groups with comparable freezing levels. On day 4, one group was injected with lenti-CRISPRv2-gRNA2, while the other group was injected with a control virus (lenti-CRIAPRv2-puro). Both groups received Rec+Ext training on day 15 and 16 (to ensure an effective extinction), followed by memory recall in context B’ on day 17 and in context C on day 18 (Fig. 5b). There was no difference in the freezing levels between the lenti-CRISPRv2-gRNA2 group and control group in all behavioral tests (Fig. 5e). When we analyzed renewal after extinction in these two groups, we found that control group showed no difference in freezing levels between context B’ and context C (Fig. 5f left), while the lenti-CRISPRv2-gRNA2 group showed higher freezing level in context C than in context B’ (T_10_ = 4.04, P < 0.01, Fig. 5f right). Thus knocking down Dnmt3a in the dDG promoted fear renewal with an extinction protocol that normally blocks fear renewal. Together with the Dnmt3a over-expression results, these results indicate that Dnmt3a regulates fear renewal in a bi-directional manner.

### Extinction training with Rec+Ext protocol prevented renewal of remote fear memory

**Figure 6:**
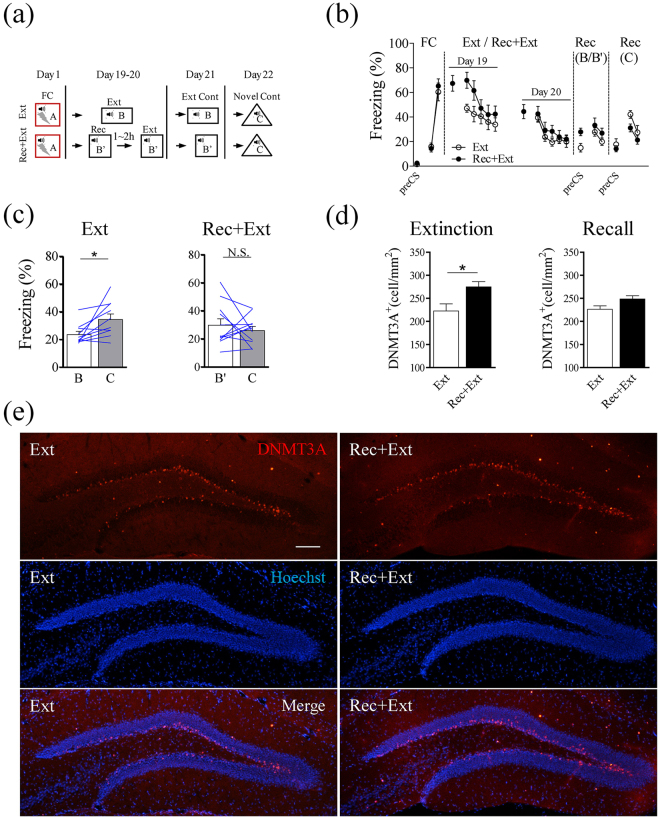
Change in the density of Dnmt3a-positive cells in the dDG after extinction of remote memory. (**a**) Experimental procedures. Day 1, fear-conditioning (FC); Day19 and Day 20, mice were given a standard extinction training (Ext, n = 10 mice), or a brief recall followed by extinction training (Rec+Ext, n = 11); Day 21, testing in the extinction context (B or B’); Day 22, testing in a novel context. (**b**) Freezing levels during the entire experiment as averages during two CS presentations. Rec+Ext/Ext training on Day19–20 were averages during four CS presentations. (**c**) Freezing levels (average during four CS presentations) of individual mouse during recall in the extinction context (**b**) and novel context (**c**). Significantly increased freezing levels was observed in the novel context in the Ext group (Left, Two-tailed paired *t*-test, T_9_ = 2.714, p < 0.05), but not in the Rec+Ext group between the two contexts (Right, Two-tailed paired *t*-test, T_10_ = 0.7432, p = 0.47). (**d**) (Left) Significant elevation in the density of Dnmt3a-positive cells in the Rec+Ext group, compared to Ext group after extinction training (n = 6 mice, Two-tailed unpaired *t*-test, T_10_ = 2.769, P < 0.05). (Right) A trend towards higher density of Dnmt3a-positive cells in the Rec+Ext group, compared to Ext group after recall in context C (n = 5 mice, Two-tailed unpaired *t*-test, T_8_ = 2.215, P = 0.058). (**e**) Sample images of Dnmt3a staining in the dDG after extinction training. Samples were collected 3 hours after extinction in context B (Ext) or B’ (Rec+Ext). Scale bars, 100 μm.

In our experiments using over-expression of Dnmt3a, we tested fear memory that was about 3 weeks old which can be regarded as remote memory. Remote memory is generally regarded as more resistant to extinction than recent memory^31,32^. The fact that renewal of this remote memory was absent with elevated Dnmt3a level suggests that Rec+Ext protocol may also be effective on remote memory. Alternatively, since Dnmt3a level was elevated for a sustained period of time in AAV-Dnmt3a over-expression (Fig. 3), this high level of Dnmt3a in the dDG might in some way “keep” the remote memory in a “recent” state (i.e., allowing this remote memory to be updated or modified as recent memory)^33^. To distinguish between these two possibilities, we fear conditioned mice on day 1, divided them into two groups based on their freezing levels, and subjected them to either Ext protocol or Rec+Ext protocol for 2 days on day 19 and 20 (Fig. 6a). Then, we tested memory recall in extinction context (B’ or B; day 21) and novel context (C; day 22) (Fig. 6a). As shown in Fig. 6b, freezing levels in both groups were significantly reduced with extinction training. When the freezing levels of each mouse was compared between extinction and novel context, we found a significant difference for the Ext group (T_9_ = 2.714, P < 0.05; Fig. 6c left) but not Rec+Ext group (T_10_ = 0.743, P = 0.47; Fig. 6c right), suggesting an absence of renewal in the Rec+Ext group. In addition, we also found a significant increase in the Dnmt3a level in the dDG in Rec+Ext group, compared to the Ext group after extinction training (T_10_ = 2.769, P < 0.05; Fig. 6d left, 6e) but only a trend towards increase after extinction training (T_8_ = 2.215, P = 0.058; Fig. 6d right). These results are consistent with the notion that Rec+Ext training is effective in preventing fear renewal, likely mediated by increasing the Dnmt3a level. However, this effect is less robust than we have found on recent memory (a few days old) using the same extinction procedure, in that: (1) we needed to give 2-day rather than 1-day extinction training (as used on recent memory) to significantly reduce freezing level; (2) although there was significant difference within group, there was no significant difference across groups (as there was for recent memory); (3) increase in Dnmt3a level was also less robust than that after extinction training for recent memory. Put together, our results suggest that an increase in Dnmt3a level might be effective in preventing fear renewal after extinction for both recent and remote memory.

## Discussion

In this study, we found: (1) Dnmt3a level was elevated in the dDG of mice underwent extinction training that resulted in the absence of fear renewal; (2) Over-expression of Dnmt3a in the dDG prevented while knockdown of Dnmt3a in the dDG promoted fear renewal; (3) renewal of both recent and remote memory can be prevented using an appropriate extinction protocol with concurrent increase in Dnmt3a level in the dDG. These findings strongly indicate that Dnmt3a regulates fear renewal after extinction. Further exploration of the underlying biological mechanism and therapeutic potential of these findings will deepen our understating of the nature of emotion-related psychiatric disorders (such as phobia, PTSD) and may also improve their treatment.

Current exposure therapy, although effective in many patients, suffers from the inability to generalize its efficacy over time, or is limited by the potential return of adverse memory in the new/novel contexts^5,34^. These limitations are caused by the context-dependent nature of extinction which is widely viewed as the biological basis of exposure therapy^5,6,27^. Thus, achieving a context-independent extinction may significantly reduce fear renewal to improve the efficacy of exposure therapy. Reduced renewal has been reported to occur after brief memory retrieval followed by extinction training^9,35^, or after extinction training in multiple contexts^36–38^. The effectiveness and consistency of these approaches are still in debate^13,14,35,39^. Our current study suggests that the effectiveness of these approaches, and ultimately the occurrence of fear renewal, is determined by the level of Dnmt3a after extinction training, especially in the dDG. This conclusion is based on our findings of bi-directional regulation of fear renewal by the Dnmt3a level.

What are the potential mechanisms underlying this Dnmt3a-depedent prevention of fear renewal? There are two potential mechanisms underlying extinction, one is erasure or updating of the formed memory^16,40,41^, and the other is the formation of a new extinction memory which suppresses or competes with the existing memory in a context-dependent manner^39^. While most studies favor the suppression mechanism in the adult, limited studies do suggest that erasure occurs in the immature animals^42–44^. Erasure of fear memory is consistent with the absence of spontaneous recovery, renewal or reinstatement observed with the Rec+Ext protocol^9^. Currently, there is no direct evidence as whether fear memory is erased after Rec+Ext protocol, and such evidence may involve direct examination of memory engram^45^. Our results showed that elevated Dnmt3a in the dDG led to a rapid and continuous reduction in freezing level with daily memory recall, regardless of the contexts where this recall took place (Fig. 3g). In contrast, in the GFP-expressing (control) mice, a continuous reduction in freezing level was seen with recall in the same contexts but recovery of freezing occurred in the novel/different text (Fig. 3g). Cued fear memory was resistant to decay after repetitive recall in the Dnmt3a KO mice and LTP was reduced in the hippocampal CA1 region^21^. Based on the above results, we propose that high Dnmt3a level makes memory more susceptible to modification, such as decay with recall and alteration in its properties (for example, its association with cue/context). On the other hand, with Dnmt3a level unaltered or reduced, memory is more resistant to modification. More specifically, we propose that if Dnmt3a level is elevated with extinction training (such as with Rec+Ext protocol), modification to the existing memory occurs and as a consequence extinction does not act as a separate mechanism or form a new memory; but if Dnmt3a level is unaltered with extinction training, a separate extinction memory is formed which acts to suppress or compete with the existing memory. This model is also consistent with findings from studies in infant/juvenile rodents that: (1) fear extinction is resistant to renewal, spontaneous recovery or reinstatement, and hence suggestive of erasure in nature^43^; (2) Dnmt3a level is high in these young rodents^46,47^. Thus, high Dnmt3a level may keep the formed memory in a dynamic, modifiable state. In this context, it is interesting to note that we were able to prevent renewal of remote fear memory with Rec+Ext protocol and this absence of fear renewal was associated with elevated Dnmt3a level in the dDG. Thus, it is worthy of further exploration of whether Dnmta3a level is a dynamic control of memory stability and modifiability which may play a critical role in the transition between recent and remote memory. It is important to note that the efficacy of extinction of remote memory is less robust than that for recent memory, which is consistent with the general findings that remote memory is more stable and less subjective to modification^33,48^. An alternative possibility is related to the epigenetic nature of DNMTs’ regulation of memory^17,49,50^. High Dnmt3a level may enhance extinction memory sufficiently to suppress/out-compete the existing fear memory in a persistent manner that is indistinguishable from erasure when measured using behavioral readout.

Another possible mechanism underlying Dnmt3a effect is altered context encoding, by which over-expression of Dnmt3a in the dDG alters how context is encoded during extinction to influence fear renewal. It is widely accepted that the dDG is more involved in the encoding of contextual information which enables the hippocampus to link context to memory^51,52^. Inactivation of dHPC pharmacologically in rats before extinction training led to renewal of fear even when tested in the extinction context^53^. Inactivating DG using optogenetic method impaired fear extinction in contextual fear conditioning^54^. Thus, increased neural activity in the HPC and dDG more specifically may prevent renewal by altering context encoding during extinction training, but whether this elevated dDG activity is sufficient is unclear. We found significant elevation of c-Fos expression in the dDG during extinction training using Rec+Ext protocol, or in mice with Dnmt3a OE in the dDG. The fact that manipulating Dnmt3a level in the dDG led to similar changes in c-Fos patterns as with Rec+Ext protocol supports the notion that Dnmt3a may alter context encoding by affecting neural activity in the dDG and that local changes in the dDG activity might be sufficient to affect renewal. Neural circuits underlying fear renewal has been found to involve ventral hippocampus, prefrontal cortex and amygdala^27,55^. Ventral hippocampal neurons projecting to both amygdala and prefrontal cortex has been shown to be critical for fear renewal^28,29^. These results were consistent with our finding of elevated density of c-Fos neurons in the ventral hippocampus but not dorsal hippocampus during fear memory renewal in AAV-GFP-expressing mice (Fig. 4a). Our findings on the elevated c-Fos density in both PrL and IL, together with elevated c-Fos density in the vHPC, are consistent with the finding of Wang *et al*.^56^ who showed that renewal of extinguished fear activates ventral hippocampal neurons projecting to the PrL and IL. Wang *et al*. interpreted their findings as that vHPC activation leads to enhanced PrL activation to promote fear and enhanced activation of local inhibitory neurons in the IL. This interpretation is in agreement with the general view that PrL and IL play different and likely opposite roles in fear related functions especially fear extinction^27^. For our c-Fos results, we did not distinguish between excitatory and inhibitory neurons. Another possible explanation is that the involvement of IL in fear renewal is different between recent and remote memory. No prior study has examined the role of IL in the renewal of remote fear memory while our c-Fos results were conducted on the renewal of remote memory. This possibility of IL may play different roles in renewal of recent and remote memory is worthy of further exploration.

Over expression of Dnmt3a in mPFC led to reduced anxiety-like behavior^23^, which could affect freezing. However, anxiety level was not altered in Dnmt3a KO mice^57^, and we found no changes in open field and elevated plus maze, two tests of innate anxiety. Nonetheless, we cannot exclude the possibility of over-expressing Dnmt3a in the dDG decreases anxiety level after traumatic experience (such as fear).

What are the potential targets of Dnmt3a? Using the next generation sequencing, genes regulated by Dnmt3a has been found to include NMDA receptors, AMPA receptors, BDNF, Fos, egr1, which are involved plasticity^58^. At this time, we cannot exclude the non-epigenetic targets of Dnmt3a. In addition, we also do not know whether the benefit of Dnmt3a OE is caused by and hence can be mimicked with enhancing Dnmt3a activity, since there is no selective enhancer of Dnmt3a at this time. Alternatively, Dnmt3a may play a structural role, such as functioning as a scaffold.

In conclusion, we have identified a key molecule in regulating fear memory renewal after extinction. Since our current results showed that renewal can be prevented for both recent and remote fear memory, we suggest that modulating Dnmt3a level/activity may have the potential to treat various chronic psychiatric diseases involving disregulated fear and/or anxiety.

## Materials and Methods

### Animals

C57BL/6 J mice were purchased from Guangdong Medical Laboratory Animal Center (China). Mice were maintained in a pathogen-free temperature-controlled (22 ± 1 °C) mouse facility on a reversed 12 h light-dark cycle (8:00 am~ 20:00 pm), with a maximum of 6 mice per cage, in Peking University Shenzhen Graduate School. Mice of 8–12 week of age were used. Recent studies showed that Dnmt3a expression is regulated by sex^59^ and age^46,47^. To exclude the impact of sex and age, we used male mice for all experiments, with every group of mice for a given set of experiment from the same butch of mice with the same age. All behavioral experiments were completed between 9:00 am to 18:00 pm. All animal experiments were performed in accordance with the ARRIVE guidelines on the Care and Use of Experimental Animals, approved by the Peking University Shenzhen Graduate School Animal Care and Use Committee.

### Fear conditioning

We have followed previously published protocols^60^, four contexts were used in fear conditioning and extinction (Fig. 1b): Context A (Coul-bourn conditioning chamber, cleaned/wiped with 70% alcohol between each animal, lighting from Coul-bourn), Context B (20 cm × 35 cm × 20 cm, rectangular box, made of Plexiglas plate, black with white floor, cleaned/wiped with the 1% acetic acid between each animal, lighting by a LED light covered with yellow shade), Context B’ (Coul-bourn conditioning chamber, switched the electric palisade to a plastic plate, cleaned/wiped with the 1% acetic acid between each animal, lighting by a LED light covered with yellow lampshade), Context C (white trihedral box, made by plexiglass plate, cleaned/wiped with 70% alcohol between each animal, lighting by a white LED light). Mice were placed in an empty cage and transferred to the training room and taken into the testing chamber. Mice that completed the tests were first transferred to a new cage and were then sent back to their home cages together after all mice were done with testing. Conditioning training took place in context A, with mice explored the chamber for 180 s before a 20 s tone (80 dB; 2700 Hz) was presented which was co-terminated with a 2 s foot shock (0.7 mA); a total of 4 pairing were given with an interval of 120 s. For recall, mice were placed into the test chamber (Context A/Context B/Context B’/Context C), allowed to explore the chamber for 180 s before a 20 s tone was delivered; tones were given for 4 times with an interval of 40 s. For extinction training, mice were allowed to explore the extinction chamber (Context B or B’) for 180 s before a 20 s tone; 20 CS was given with an interval of 20 s. Mice were kept in the chamber for 120 s after training was completed. Freezing levels were analyzed using Coul-bourn analyze system (USA) by computer, the threshold of freezing was set to as at least more than 1 s without any movement.

### Recombinant adeno-associated virus (AAV)

An AAV vector expressing eGFP under CaMKIIα promoter (pAAV-CaMKIIα-eGFP) was purchased from Addgene (#50469). Mouse Dnmt3a (GenBank: AF068625.2) was cloned from mouse brain cDNA, inserted into pAAV-CaMKIIα-eGFP using restriction enzyme EcoR I and Hind III, then sequenced by BGI (China). Viral particles were produced as described in the rAAV Production protocol of AAV- DJ/8 Helper Free Packaging System (Cell Biolabs, USA). pAAV-CaMKIIα-eGFP-Dnmt3a, pAAV-DJ/8^61^ and pHelper (Cell Biolabs, VPK-400-DJ-8) were co-transfected (1.5:1:1) into HEK293T at 70% confluence using the lipofectin-mediated transfection method (Lipofectamine™ LTX, Invitrogen). Cells were incubated for 72 hours at 37 °C with 5% CO_2_. After 72 hours, cells were collected to purify virus using an AAV purification kit (Biomiga, USA). Purified AAV particles were titered for genome content using real-time qPCR (Promega, USA). Titers were 6 × 10^12^ GC (Genome Copy) per milliliter. Control virus, rAAV-CaMKIIα-GFP, was obtained from HANBIO company (China), with a titer of 1.3 × 10^12^ GC.

### Recombinant lentivirus

We used a lentivirus vector LentiCRISPRv2 from Addgene (#52961), inserted the guide RNA sequence as the lentiGuide oligo cloning protocol^62^. The Dnmt3a CRISPR Guide RNA sequences were designed by Feng Zhang’s laboratory at the Broad institute to selectively target Dnmt3a gene in the genome, and purchased from GENEWIZ (China). CRISPR/Cas9-Mediated Gene Knock-Down via Lentivirus has been used in neuron system^63^. The lentivirus was produced as described previously^64^. Lenti-CRISPRv2-gRNA, pMD2.G and psPAX2 was co-transfected (2: 1.5: 2) into HEK293T cells at 70% confluence using lipofectin-mediated transfection method (Lipofectamine™ LTX, Invitrogen, USA). Cells were incubated for 72 hours at 37 °C with 5% CO_2_. After 72 hours, culture medium was collected and lentivirus were purified using a lentivirus purification kit (Biomiga, USA). A control virus, Lenti-CRISPRv2-puro, was obtained from HANBIO (China).

### Stereotaxic surgery

All surgeries were performed under stereotaxic guidance. Mice were anaesthetized using isoflurane. Virus was injected using a 5 microliter microsyringe (Hamilton) through a 33 G needle, A microsyringe pump controller (KD Scientific) were used to control the speed of injection. rAAV sand lentivirus were injected into the dDG of hippocampus using the following coordinates, from Bregma: AP = −2.1 mm; ML = ±1.4 mm; DV = −1.9 mm. Injection speed was 150 nl/min (0.8~1.0 μl for AAV-Dnmt3a, 0.3 μl for AAV-GFP, 1.0~1.5 μl for lentivirus). The capacity of AAV was about 5k bp, while the insert DNA of pAAV-CaMKIIα-eGFP-Dnmt3a is 4.7k, hence very close to this limit. As a result, the infection efficiency of the AAV-Dnmt3a virus is lower than that of the control virus when similar titers were used. To ensure the efficacy of infection, we had used 3 times higher titer for the AAV-Dnmt3a virus than the control virus, and the control virus was diluted 5 times before use. The injection needle was kept in place for at least 180 s to allow sufficient diffusion.

### Immunohistochemistry

Mice were anesthetized with pentobarbital sodium, perfused with 4% paraformaldehyde and PBS through the heart. Brains were removed, post-fixed in the 4% PFA at 4 °C overnight and placed in PBS containing 30% sucrose. For quick-freeze by liquid nitrogen in OCT, slices were cut at 30 μm. Brain sections for immunofluorescence were treated with 0.5% Triton X-100, 10% goat serum and 0.2% skim milk powder in PBS for 1 hour at room temperature. Section were incubated with primary antibody (anti-DNMT3A (Rabbit), 1:1000, CST, #3598, USA) overnight at room temperature, washed with PBS three time (each for 10 min), incubated with secondary antibody (goat anti-Rabbit 488/546, 1:400, Invitrogen, A11008 or A-11035, USA) for 1 hour in the dark at room temperature, and washed three times in PBS (each for 10 min). Finally, sections were incubated with Hoechst for 5 min and mounted on glass slides. For c-Fos immunohistochemistry, sections were treated with 3% H_2_O_2_ in PBS for 30 min at room temperature, permeabilized with 0.5% TritonX-100 in PBS for 10 min at room temperature, treated using the protocol of Mouse on Mouse Basic Kit (VECTOR, BMK-2202, USA). c-Fos antibody (anti-c-Fos (mouse) 1:1000; Abcam ab208942, USA) was used. Cells were stained using the DAB method (VECTOR, SK-4105, USA) and mounted on glass slides.

For double immunostaining of Dnmt3a and NeuN, we used anti-DNMT3A (CST, #3598, 1:1000). anti-NeuN (mouse) (Milipore, MAB377, 1:5000) as primary antibodies, and secondary antibody from Invitrogen (goat anti-Rabbit 488, A11008, 1:400; goat anti-Mouse 546, A-11003, 1:400). For double immunostaining of Dnmt3a and GAD67 (using GAD67-GFP mouse), we used anti-DNMT3A (CST, #3598, 1:1000) with secondary antibody from Invitrogen (Goat anti-Rabbit IgG 546, A-11035, 1:400). For double immunostaining of Dnmt3a and GFAP, we used anti-DNMT3A (CST, #3598, 1:1000), anti-GFAP (mouse) (Millipore, MAB360, 1:500), with secondary antibody from Invitrogen (goat anti-Rabbit 488, A11008, 1:400; goat anti-Mouse 546, A11003, 1:400).

### Cell counting

c-Fos IHC images (DAB method) were taken on inverted fluorescence microscope (IX73, Olympus, Japan) with a 4× objective was used, positive cells were counted by using Image J based on The Mouse Brain in Stereotaxic Coordinates^65^. More than three sections for every region were taken for each mouse. Every experiment on c-Fos IHC was repeated twice for one brain simple. For Ext and Rec+Ext group Dnmt3a immunofluorescence, images were taken on inverted fluorescence microscope with a 10× objective was used. Positive cells merged with Hoechst were counted by using Image J, and sample images were taken using confocal microscope (LSM510 META, ZEISS, Germany). For Dnmt3a double staining (NeuN, GAD67, GFAP) all images were taken using a confocal microscope with a 20× objective. Co-localization of Dnmt3a- and NeuN-positive cells was counted using Image J.

### Open field test

Mice were habituated in the testing room (lighting 10 Lux) for 1–2 hours before behavioral testing. Each mouse was placed in the open field apparatus (50 cm × 50 cm × 50 cm). Total distance, distance in the center area and time in the center area during a period of 15 min were analyzed using ANY-maze software.

### Elevated plus maze test

The apparatus consists of two open arms: two closed arms with a wall (30 cm × 5 cm × 15 cm) and a central area (5 cm × 5 cm). This apparatus was placed 35 cm above the floor. Mice were habituated in the testing room (the light is 50 Lux) for 1–2 hours before behavioral test. Each mouse was placed in the center platform and faced to the open arms. Time and distance in the open arms and closed arms for during a 5 min period was analyzed using ANY-maze software.

Mice used for OF and EPM were from different cohorts that those used in behavioral testing and did not undergo any extinction test, but only to test the potential effect of AAV expression.

### Data analysis

Behavioral data were recorded and analyzed using softwares from Coul-bourn(USA), and ANY-maze Behavioural tracking software(USA). Mice without any virus expression in the dDG were excluded from data analysis and removed from data set. Statistical analysis was performed using paired/unpaired *t*-test and Two-way ANOVA (GraphPad Prism software), as noted. Results were expressed as mean ± SEM. P < 0.05 was considered as significant.

## Electronic supplementary material

Supplementary information

## References

[1] Vervliet B, Craske MG, Hermans D (2013). Fear extinction and relapse: state of the art. Annu Rev Clin Psychol.

[2] Rachman S (1989). The Return of Fear - Review and Prospect. Clin Psychol Rev.

[3] Mineka S, Mystkowski JL, Hladek D, Rodriguez BI (1999). The effects of changing contexts on return of fear following exposure therapy for spider fear. J Consult Clin Psychol.

[4] Rodriguez BI, Craske MG, Mineka S, Hladek D (1999). Context-specificity of relapse: effects of therapist and environmental context on return of fear. Behav Res Ther.

[5] Podlesnik CA, Kelley ME, Jimenez-Gomez C, Bouton ME (2017). Renewed behavior produced by context change and its implications for treatment maintenance: A review. J Appl Behav Anal.

[6] Vervliet B, Baeyens F, Van den Bergh O, Hermans D (2013). Extinction, generalization, and return of fear: a critical review of renewal research in humans. Biol Psychol.

[7] Bouton ME, King DA (1983). Contextual control of the extinction of conditioned fear: tests for the associative value of the context. J Exp Psychol Anim Behav Process.

[8] Bouton ME (2004). Context and behavioral processes in extinction. Learn Memory.

[9] Monfils MH, Cowansage KK, Klann E, LeDoux JE (2009). Extinction-Reconsolidation Boundaries: Key to Persistent Attenuation of Fear Memories. Science.

[10] Baker KD, McNally GP, Richardson R (2013). Memory retrieval before or after extinction reduces recovery of fear in adolescent rats. Learn Memory.

[11] Ponnusamy R (2016). Retrieval and Reconsolidation Accounts of Fear Extinction. Front Behav Neurosci.

[12] Schiller D (2010). Preventing the return of fear in humans using reconsolidation update mechanisms. Nature.

[13] Ishii D (2012). No erasure effect of retrieval-extinction trial on fear memory in the hippocampus-independent and dependent paradigms. Neurosci Lett.

[14] Ishii D (2015). An isolated retrieval trial before extinction session does not prevent the return of fear. Behav Brain Res.

[15] Kindt M, Soeter M (2013). Reconsolidation in a human fear conditioning study: A test of extinction as updating mechanism. Biological Psychology.

[16] Graff J (2014). Epigenetic priming of memory updating during reconsolidation to attenuate remote fear memories. Cell.

[17] Miller CA, Sweatt JD (2007). Covalent modification of DNA regulates memory formation. Neuron.

[18] Meadows JP (2015). DNA methylation regulates neuronal glutamatergic synaptic scaling. Sci Signal.

[19] Day JJ, Sweatt JD (2010). DNA methylation and memory formation. Nat Neurosci.

[20] Bird A (2002). DNA methylation patterns and epigenetic memory. Genes Dev.

[21] Morris MJ, Adachi M, Na ES, Monteggia LM (2014). Selective role for DNMT3a in learning and memory. Neurobiol Learn Mem.

[22] Mitchnick KA, Creighton S, O’Hara M, Kalisch BE, Winters BD (2015). Differential contributions of de novo and maintenance DNA methyltransferases to object memory processing in the rat hippocampus and perirhinal cortex–a double dissociation. Eur J Neurosci.

[23] Elliott E (2016). Dnmt3a in the Medial Prefrontal Cortex Regulates Anxiety-Like Behavior in Adult Mice. J Neurosci.

[24] LaPlant Q (2010). Dnmt3a regulates emotional behavior and spine plasticity in the nucleus accumbens. Nat Neurosci.

[25] Marin MF (2017). Skin Conductance Responses and Neural Activations During Fear Conditioning and Extinction Recall Across Anxiety Disorders. Jama Psychiat.

[26] Haaker J (2015). Deficient inhibitory processing in trait anxiety: Evidence from context-dependent fear learning, extinction recall and renewal. Biol Psychol.

[27] Maren S, Phan KL, Liberzon I (2013). The contextual brain: implications for fear conditioning, extinction and psychopathology. Nat Rev Neurosci.

[28] Jin, J. J. & Maren, S. Fear renewal preferentially activates ventral hippocampal neurons projecting to both amygdala and prefrontal cortex in rats. *Sci Rep-Uk* **5** (2015). 10.1038/srep08388 PMC4323647 25669753

[29] Xu C (2016). Distinct Hippocampal Pathways Mediate Dissociable Roles of Context in Memory Retrieval. Cell.

[30] Swiech L (2015). *In vivo* interrogation of gene function in the mammalian brain using CRISPR-Cas9. Nat Biotechnol.

[31] Frankland PW, Bontempi B (2005). The organization of recent and remote memories. Nat Rev Neurosci.

[32] Norris D (2017). Short-term memory and long-term memory are still different. Psychological bulletin.

[33] Tsai LH, Graff J (2014). On the resilience of remote traumatic memories against exposure therapy-mediated attenuation. Embo Rep.

[34] Boschen MJ, Neumann DL, Waters AM (2009). Relapse of successfully treated anxiety and fear: theoretical issues and recommendations for clinical practice. Aust Nz J Psychiat.

[35] Auber A, Tedesco V, Jones CE, Monfils MH, Chiamulera C (2013). Post-retrieval extinction as reconsolidation interference: methodological issues or boundary conditions?. Psychopharmacology.

[36] Balooch SB, Neumann DL, Boschen MJ (2012). Extinction treatment in multiple contexts attenuates ABC renewal in humans. Behav Res Ther.

[37] Bustamante J, Uengoer M, Thorwart A, Lachnit H (2016). Extinction in multiple contexts: Effects on the rate of extinction and the strength of response recovery. Learning & behavior.

[38] Gunther LM, Denniston JC, Miller RR (1998). Conducting exposure treatment in multiple contexts can prevent relapse. Behav Res Ther.

[39] Bouton, M. E. Context, ambiguity, and unlearning: Sources of relapse after behavioral extinction. *Biol Psychia*t **52**, 976–986, Pii S0006–3223(02)01546-9 10.1016/S0006-3223(02)01546-9 (2002). 10.1016/s0006-3223(02)01546-9 12437938

[40] Quirk GJ (2010). Erasing Fear Memories with Extinction Training. Journal Of Neuroscience.

[41] Oyarzun, J. P. *et al*. Updating Fearful Memories with Extinction Training during Reconsolidation: A Human Study Using Auditory Aversive Stimuli. *Plos One* **7** (2012). 10.1371/journal.pone.0038849 PMC3387215 22768048

[42] Kim JH, Richardson R (2007). A developmental dissociation of context and GABA effects on extinguished fear in rats. Behavioral neuroscience.

[43] Gogolla N, Caroni P, Luthi A, Herry C (2009). Perineuronal Nets Protect Fear Memories from Erasure. Science.

[44] Park CHJ, Ganella DE, Kim JH (2017). A dissociation between renewal and contextual fear conditioning in juvenile rats. Dev Psychobiol.

[45] Roy DS, Muralidhar S, Smith LM, Tonegawa S (2017). Silent memory engrams as the basis for retrograde amnesia. Proceedings of the National Academy of Sciences of the United States of America.

[46] Feng J, Chang H, Li E, Fan GP (2005). Dynamic expression of de novo DNA methyltransferases Dnmt3a and Dnmt3b in the central nervous system. J Neurosci Res.

[47] Simmons RK, Stringfellow SA, Glover ME, Wagle AA, Clinton SM (2013). DNA methylation markers in the postnatal developing rat brain. Brain Res.

[48] Frankland PW (2006). Stability of recent and remote contextual fear memory. Learn Mem.

[49] Kennedy AJ, Sweatt JD (2016). Drugging the methylome: DNA methylation and memory. Critical reviews in biochemistry and molecular biology.

[50] Heyward FD, Sweatt JD (2015). DNA Methylation in Memory Formation: Emerging Insights. The Neuroscientist: a review journal bringing neurobiology, neurology and psychiatry.

[51] Kesner RP (2013). An analysis of the dentate gyrus function. Behav Brain Res.

[52] Kheirbek MA (2013). Differential control of learning and anxiety along the dorsoventral axis of the dentate gyrus. Neuron.

[53] Corcoran KA, Desmond TJ, Frey KA, Maren S (2005). Hippocampal inactivation disrupts the acquisition and contextual encoding of fear extinction. J Neurosci.

[54] Bernier BE (2017). Dentate Gyrus Contributes to Retrieval as well as Encoding: Evidence from Context Fear Conditioning, Recall, and Extinction. Journal Of Neuroscience.

[55] Chen W, Wang Y, Wang X, Li H (2017). Neural circuits involved in the renewal of extinguished fear. IUBMB life.

[56] Wang Q, Jin J, Maren S (2016). Renewal of extinguished fear activates ventral hippocampal neurons projecting to the prelimbic and infralimbic cortices in rats. Neurobiol Learn Mem.

[57] Morris MJ, Na ES, Autry AE, Monteggia LM (2016). Impact of DNMT1 and DNMT3a forebrain knockout on depressive- and anxiety like behavior in mice. Neurobiol Learn Mem.

[58] Colquitt BM, Markenscoff-Papadimitriou E, Duffie R, Lomvardas S (2014). Dnmt3a regulates global gene expression in olfactory sensory neurons and enables odorant-induced transcription. Neuron.

[59] Nugent BM (2015). Brain feminization requires active repression of masculinization via DNA methylation. Nat Neurosci.

[60] Curzon, P., Rustay, N. R. & Browman, K. E. In *Methods of Behavior Analysis in Neuroscience Frontiers in Neuroscience* (ed. J. J. Buccafusco) (2009).

[61] Grimm D (2008). *In vitro* and *in vivo* gene therapy vector evolution via multispecies interbreeding and retargeting of adeno-associated viruses. Journal of virology.

[62] Sanjana NE, Shalem O, Zhang F (2014). Improved vectors and genome-wide libraries for CRISPR screening. Nature methods.

[63] Straub C, Granger AJ, Saulnier JL, Sabatini BL (2014). CRISPR/Cas9-mediated gene knock-down in post-mitotic neurons. Plos One.

[64] Nasri M, Karimi A, Allahbakhshian Farsani M (2014). Production, purification and titration of a lentivirus-based vector for gene delivery purposes. Cytotechnology.

[65] Paxinos, G., Franklin, K. B. J. & Franklin, K. B. J. *The mouse brain in stereotaxic coordinates*. 2nd edn, (Academic Press, 2001).

