# Supplementary material for: Dnmt3a in the dorsal dentate gyrus is a key regulator of fear renewal
Source: Sci Rep. 2018 Mar 23;8:5093. doi: 10.1038/s41598-018-23533-w (PMC5865109; doi:10.1038/s41598-018-23533-w)
Supplement: Supplementary file 1 — Supplementary information [file 41598_2018_23533_MOESM1_ESM.doc]

**Dnmt3a in the dorsal dentate gyrus is a key regulator of fear renewal**

Supplemental Information

**Authors:** Zhiting Gong and Qiang Zhou*

School of Chemical Biology and Biotechnology, Peking University Shenzhen Graduate School, Shenzhen, China


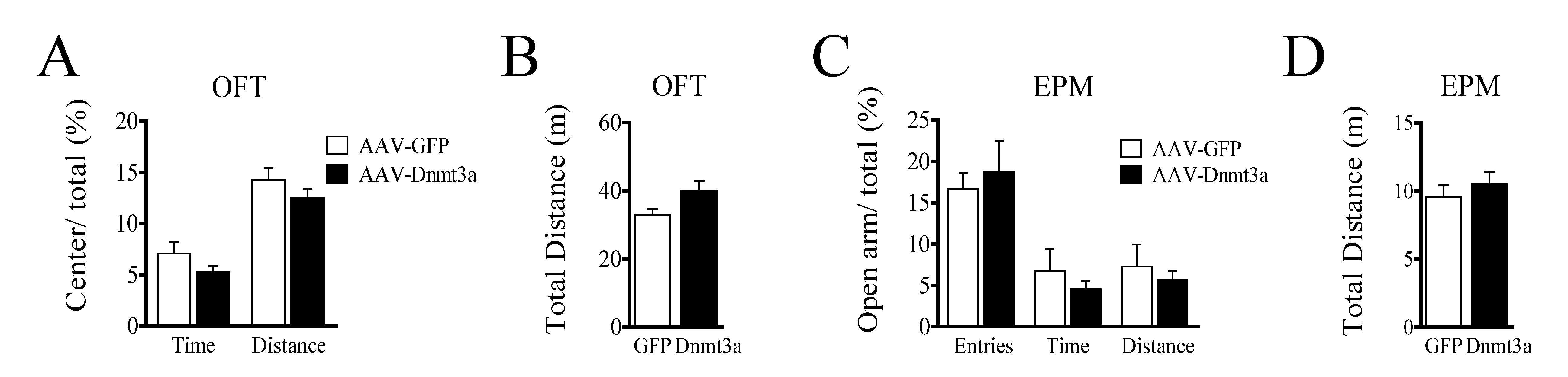


Figure S1. Test of innate anxiety level after AAV infection.

Mice with infection of either AAV-GFP or AAV-Dnmt3a did not show any difference in the OFT, including center time and center distance (A) (Two-way ANOVA, F_1, 44_=3.56, P = 0.07). But there was a trend towards increase in total distance (B) (Two-tailed unpaired *t*-test, T_22_ = 2.03, P = 0.054) (AAV-GFP, n = 12 mice; AAV-Dnmt3a, n = 12 mice). AAV-GFP and AAV-Dnmt3a mice did not show any significant difference in EPM including entries, times and distance in the open arm (C) (Two-way ANOVA, F _1, 60_ = 0.09, P = 0.77), or total distance (D) (Two-tailed unpaired *t*-test, T_20_ = 0.75, P = 0.46) (AAV-GFP, n = 10 mice; AAV-Dnmt3a, n = 12 mice).


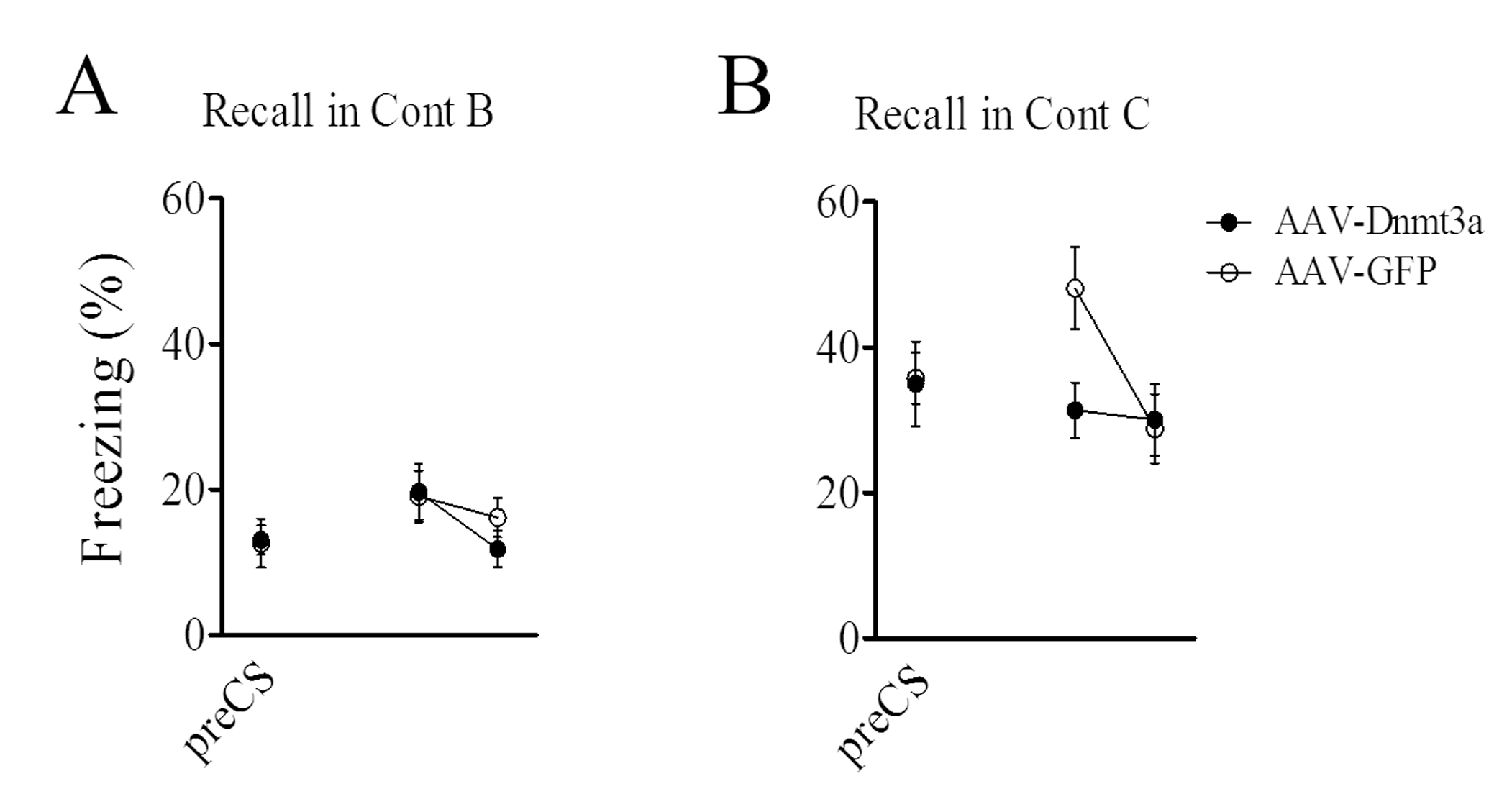


Figure S2. Behavioral results corresponded to Figure 4A showing freezing levels during recall prior to IHC. Freezing levels were averages during two CS presentations.


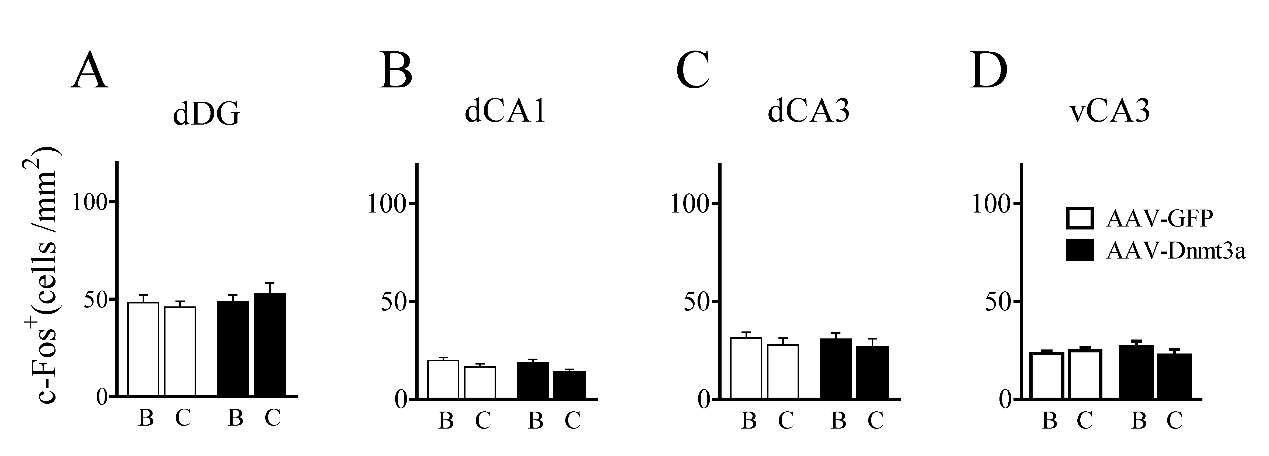


Figure S3. The patterns of c-Fos positive cells in mouse brains with overexpression of Dnmt3a.

Quantification of c-Fos in each section from mice injected with AAV-GFP or AAV-Dnmt3a. Sections were collected 1.5 hours after recall in context B (AAV-GFP, n = 12 mice; AAV-Dnmt3a, n= 10 mice), or context C (AAV-GFP, n = 10 mice; AAV-Dnmt3a, n = 10 mice) No significant difference was found in these brain regions (Two-way ANOVA, repeated measures with Bonferroni post-test, context B vs. context C).

A

B


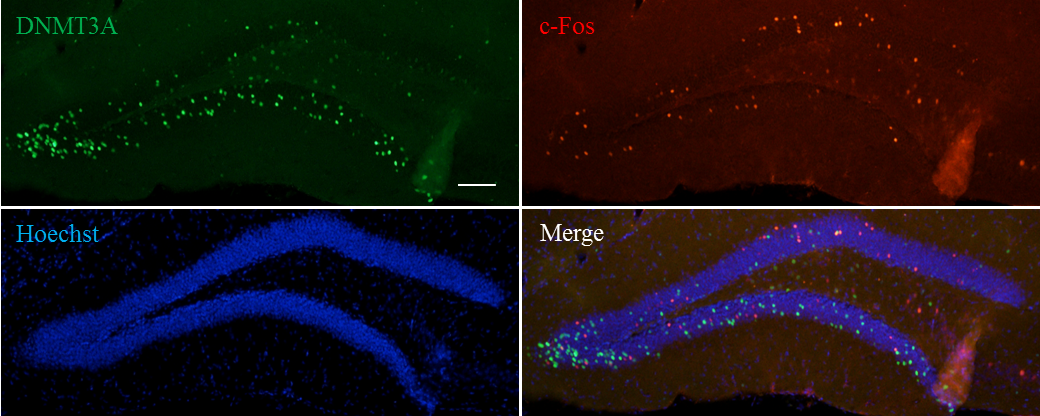


C


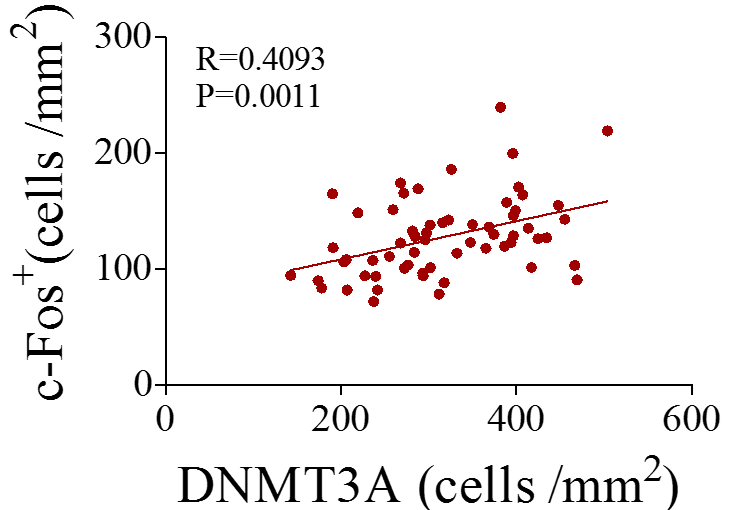


Figure S4. Behavioral results, c-Fos staining corresponding to Figure 4B and 4C.

(A) Freezing levels during the entire experiment, as averages during two CS presentations.

(B) Double immunostaining of Dnmt3a and c-Fos. Scale bars, 100 μm.

(C) Correlation between density of c-Fos-positive cells and Dnmt3a-positive cells. Each point represents one brain section.


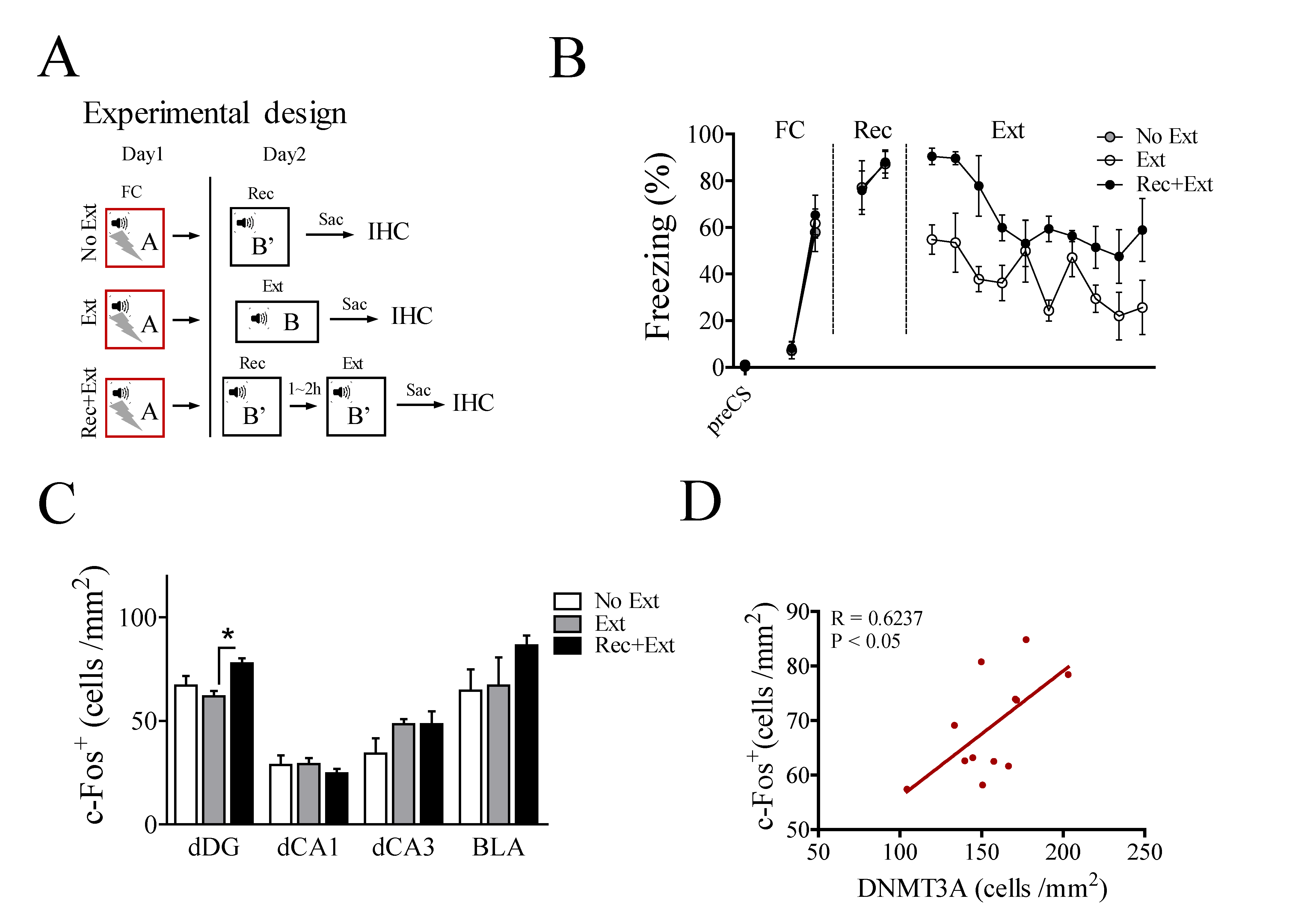


Figure S5. Elevated c-Fos staining was observed in Rec+Ext group in the dDG.

(A) Experimental design. Mice were fear-conditioned (FC) on Day 1, underwent Ext (n = 4 mice), Rec+Ext (n = 4 mice) or No Ext (4 CS recall, n = 4 mice) on Day 2. Mouse brains were collected 3 hours after behavioral training on Day 2.

(B) Freezing levels during the entire experiment. Freezing levels after fear conditioning and recall after extinction were averages during two CS presentations.

(C) Quantification of the density of c-Fos positive cells in each brain section from mice used in (A). No significant difference was found in dCA1 (One-way ANOVA, F_2, 9_ = 0.49, p = 0.63), dCA3 (One-way ANOVA, F_2, 9_ = 1.99, p = 0.19), BLA (One-way ANOVA, F_2, 9_= 1.38, p = 0.30). Elevated c-Fos density was found in Rec+Ext group in dDG (One-way ANOVA, F_2, 9_ = 5.61, p < 0.05) (N = 4 mice, at least 3 sections per mouse for every brain region).

(D) Correlation between the density of c-Fos-positive and Dnmt3a-positive cells in the dDG (Dnmt3a result was shown in Figure1E left).

A

| pcDNA3.0-Dnmt3a  ctrl | pcDNA3.0-Dnmt3a  guide RNA1 | pcDNA3.0-Dnmt3a  guide RNA2 | pcDNA3.0-Dnmt3a  guide RNA3 |
| --- | --- | --- | --- |
| pcDNA3.0-Dnmt3a  ctrl | pcDNA3.0-Dnmt3a  guide RNA1 | pcDNA3.0-Dnmt3a  guide RNA2 | pcDNA3.0-Dnmt3a  guide RNA3 |
| pcDNA3.0-Dnmt3a  ctrl | pcDNA3.0-Dnmt3a  guide RNA1 | pcDNA3.0-Dnmt3a  guide RNA2 | pcDNA3.0-Dnmt3a  guide RNA3 |


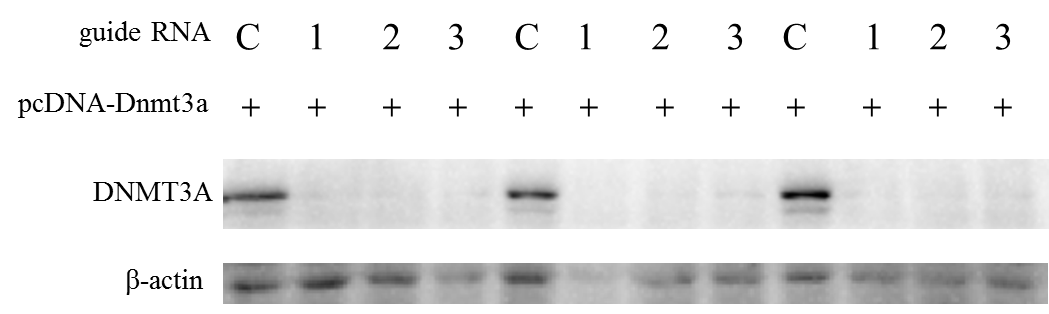


Figure S6. Testing the efficiency of guide RNA sequence. pcDNA-Dnmt3a and ctrl/pLentiCRISPRv2-guide RNA were co-transfected into HEK293T cells in 12 well as shown in (A), the ctrl/pLentiCRISPRv2-guide RNA was re-transfected 6 hours after first co-transfection. Forty-eight hours later, cells were collected for western blot analysis. Anti-DNMT3a (1:1000, Novus, USA), Anti-β-actin (1:3000, Santa Cruz, USA) were used for western blot (B). DNMT3A and β-actin bands were cut from the same gel, and each band corresponded to the one well shows in (A) (total protein from cells).

Supplementary Table. Guide RNA sequence for construction lentivirus

| gRNA 1 | AAACCCGTCAGCGACCCATGCCAAC |
| --- | --- |
| gRNA 2 | AAACTCTACGAAGTCCTCCAGGTGC |
| gRNA 3 | AAACGCACGGCTGCTGGCCACCTAC |
